# Circadian disruption and sleep disorders in neurodegeneration

**DOI:** 10.1186/s40035-023-00340-6

**Published:** 2023-02-13

**Authors:** Yun Shen, Qian-kun Lv, Wei-ye Xie, Si-yi Gong, Sheng Zhuang, Jun-yi Liu, Cheng-jie Mao, Chun-feng Liu

**Affiliations:** 1 Department of Neurology and Clinical Research Center of Neurological Disease, The Second Affiliated Hospital of Soochow University, Suzhou, 215004 China; 2 Jiangsu Key Laboratory of Neuropsychiatric Diseases and Institute of Neuroscience, Soochow University, Suzhou, 215123 China; 3 Department of Psychiatry, Faculty of Medicine, The Chinese University of Hong Kong, Shatin, N. T., Hong Kong SAR China; 4 Department of Neurology, Dushu Lake Hospital Affiliated to Soochow University, Suzhou, 215125 China

**Keywords:** Circadian disruption, Sleep disorder, Parkinson’s disease, Alzheimer’s disease

## Abstract

Disruptions of circadian rhythms and sleep cycles are common among neurodegenerative diseases and can occur at multiple levels. Accumulating evidence reveals a bidirectional relationship between disruptions of circadian rhythms and sleep cycles and neurodegenerative diseases. Circadian disruption and sleep disorders aggravate neurodegeneration and neurodegenerative diseases can in turn disrupt circadian rhythms and sleep. Importantly, circadian disruption and various sleep disorders can increase the risk of neurodegenerative diseases. Thus, harnessing the circadian biology findings from preclinical and translational research in neurodegenerative diseases is of importance for reducing risk of neurodegeneration and improving symptoms and quality of life of individuals with neurodegenerative disorders via approaches that normalize circadian in the context of precision medicine. In this review, we discuss the implications of circadian disruption and sleep disorders in neurodegenerative diseases by summarizing evidence from both human and animal studies, focusing on the bidirectional links of sleep and circadian rhythms with prevalent forms of neurodegeneration. These findings provide valuable insights into the pathogenesis of neurodegenerative diseases and suggest a promising role of circadian-based interventions.

## Background

Circadian rhythms are physiological and behavioral oscillations that manifest at every level of tissue from gene expression to interorgan functional coordination, which are regulated by an endogenous process with a periodicity of ~ 24 h that persists in the absence of environmental cues [1]. The circadian system in the brain influences many important functions including sleep-wake cycle, temperature, eating, and social interaction, and is believed to be paramount for maintaining synchrony between internal physiology, behavior, and the stimulus from the environment. The importance of such system lies in several aspects of metabolic, cognitive, immunological and oncogenic processes in the brain. Sleep-wake behavior is the most established and widely recognized sign of circadian system [2]. According to the different manifestations of electroencephalography (EEG), eyes movements and electromyography (EMG), sleep is divided into two different phases: rapid eye movement (REM) and non-rapid eye movement (NREM) sleep. Disruptions of the circadian rhythm can profoundly affect health in a wide range of functions, including sleep, alertness, cognition, psychology, motor control and metabolism [3], and have been correlated with several health problems such as neurodegenerative disorders [2].

Neurodegenerative disorders, especially Parkinson's disease (PD), Alzheimer’s disease (AD) and Huntington’s disease (HD), involve a wide range of clinical symptoms (e.g., motor and non-motor symptoms), many of which exhibit diurnal and nocturnal variations in frequency and intensity. The prevalence of circadian and sleep dysfunction varies greatly across neurodegenerative diseases (Table 1) and plays an important role in differential diagnosis. Meanwhile, circadian and sleep dysfunctions are not only a consequence of neurodegeneration but may also play a causative role. In other words, dysregulated circadian rhythm and sleep could predispose disease onset or exacerbate disease progression, in which circadian dysfunction and neurodegeneration form a detrimental, self-perpetuating transcriptional-translation feedback loop (TTFL) [4]. A deeper understanding of the relationship between circadian rhythms and neurodegeneration is essential for early identification and management of neurodegenerative diseases.

**Table 1:** Prevalence of sleep disorders in neurodegenerative diseases

	Sleep-wake disorder		Parasomnia			SDB	RLS
	Insomnia	EDS	REM parasomnia		NREM parasomnia		
			RBD	Nightmares			
PD	32%–44% [241–243]	21%–76% [27, 243]	39%–46% [242, 243]	17.2%–30% [241, 244]	Sleepwalking: (0.9%–1.8%) [158, 241] Night terror: 3.9% [241] NREM arousal-related disorder: 10.3% [245]	27.6%–48% [32, 246]	14% [57]
MSA	19% [247]	28% [248]	88% [249]		–	Stridor: 30%–42% OSA:15%–37% [250]	4.7%–28% [251]
DLB	26%–75% [252]	11%–100% [252]	76% [112]	83% [252]	–	34.8%–60% [253]	–
PDD	72% [252]	83% [252]	17% [252]	78% [252]			
FTD	48% [254]	64% [254]	Rare [254]	Rare [255]	–	68% [254]	8% [254]
CBD	Rare [256, 257]	–	14.3% [258]	–	–	Rare [259]	Rare [259]
PSP	60% [260]	60% [260]	11.4%–28% [258, 261]	–	–	55% [262]	3.7%–58% [263]
AD	40% [48]	45% [264]	Rare [265]	–	–	15%–54% [56, 63, 64]	4%–6% [55, 56]
HD	25%–51% [266, 267]	35.4%–50% [268, 269]	12%–25.8% [269, 270]	22.5% [269]	–	30.8% [271]	15.4% & one family case report [60, 271]

*EDS* excessive daytime sleepiness; *RBD* rapid eye movement (REM) sleep behavior disorder; *NREM* non-REM; *SDB* sleep-disordered breathing; *RLS* restless leg syndrome; *PD* Parkinson’s disease; *MSA* multiple system atrophy; *DLB* dementia with lewy bodies; *FTD* frontotemporal dementia; *CBD* corticobasal degeneration; *PSP* progressive supranuclear palsy; *AD* Alzheimer’s disease; *HD* Huntington’s disease; *OSA* obstructive sleep apnea

In this review, we summarize the existing literature on the circadian/sleep disruption in neurodegenerative diseases, focusing on the bidirectional links of circadian rhythm and sleep disruptions with neurodegeneration, based on molecular changes, clinical symptom variations, as well as the available treatment options.

## Mechanisms underlying circadian rhythm and sleep-wake activity

### Circadian rhythms

#### Key neuroanatomical pathways of the circadian system

**Fig. 1:**
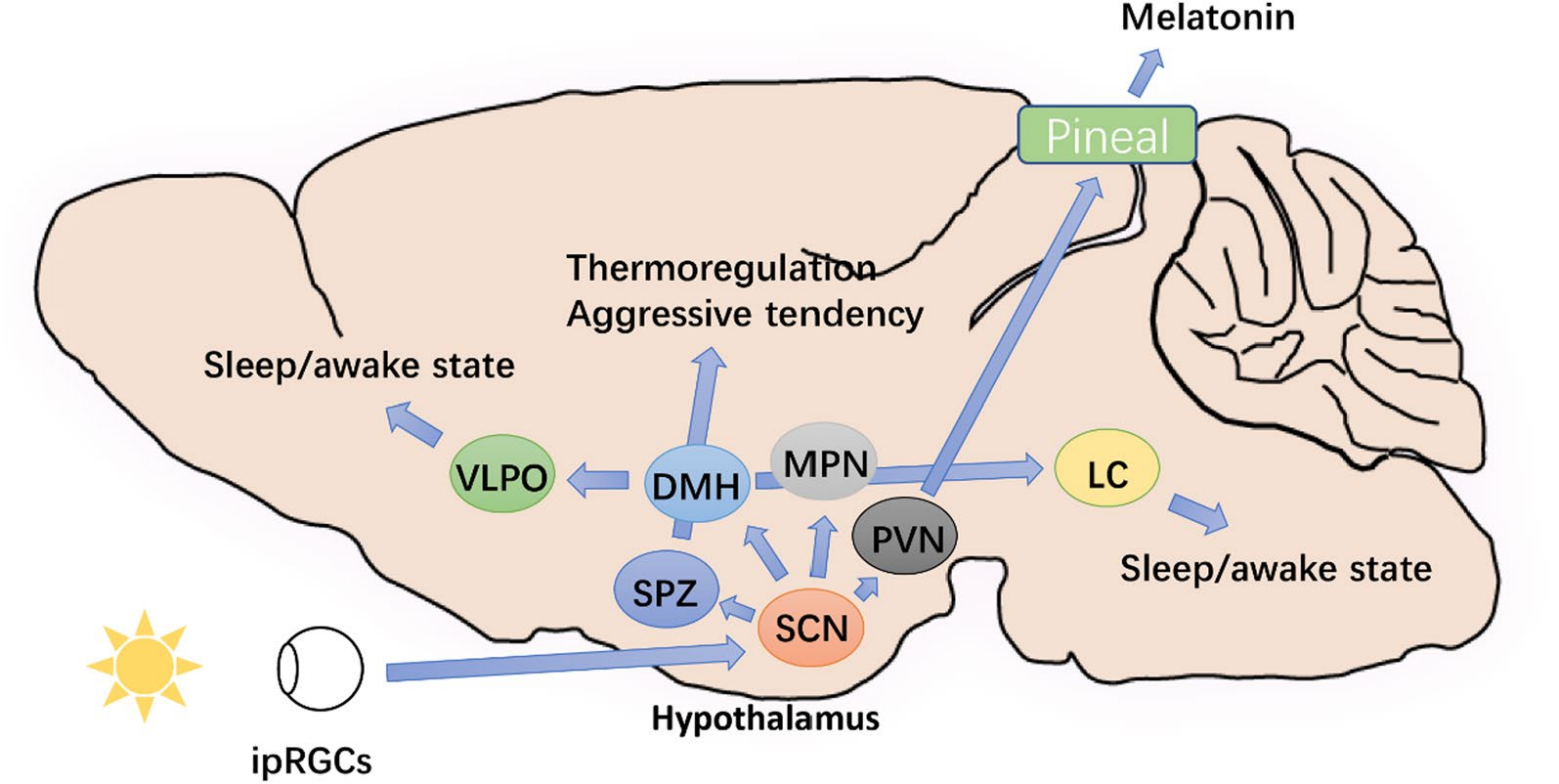
Key neuroanatomical pathways of the circadian system. (1) SCN indirectly regulates melatonin release from the pineal gland by projecting light signals to PVN. (2) DMH receives light signals from SCN and then projects them to LC and VLPO, which in turn regulates sleep/awake activity. (3) SCN regulates  thermoregulation and aggressive tendency by DMH through SPZ or not through SPZ. *DMH* dorsomedial hypothalamic nucleus; *SPZ* subparaventricular zone;

Circadian rhythms govern a wide range of physiological and behavioral processes in organisms, and can be observed at the central and the peripheral. Suprachiasmatic nucleus (SCN), which consists of thousands of neurons that exhibit self-sustaining and synchronous circadian rhythms in their electrical activity, is an important basis for behavioral and physiological rhythms. In humans, the regulation of circadian rhythm begins with the propagation of light information. Light is first detected by intrinsically photoreceptive retinal ganglion cells (ipRGCs) and then delivered to the SCN; the SCN receives and encodes light information, and then synchronizes circadian oscillations and projects signals to other brain regions [5] (Fig. 1).

The signals encoded by the SCN are mainly projected to the hypothalamus, which acts as a mediator to regulate the specific circadian rhythm. These brain regions include paraventricular nucleus (PVN), dorsomedial hypothalamic nucleus, subparaventricular zone, and medial preoptic nucleus (MPN) [6] (Fig. 1).

#### Schema of the circadian clock system

**Fig. 2:**
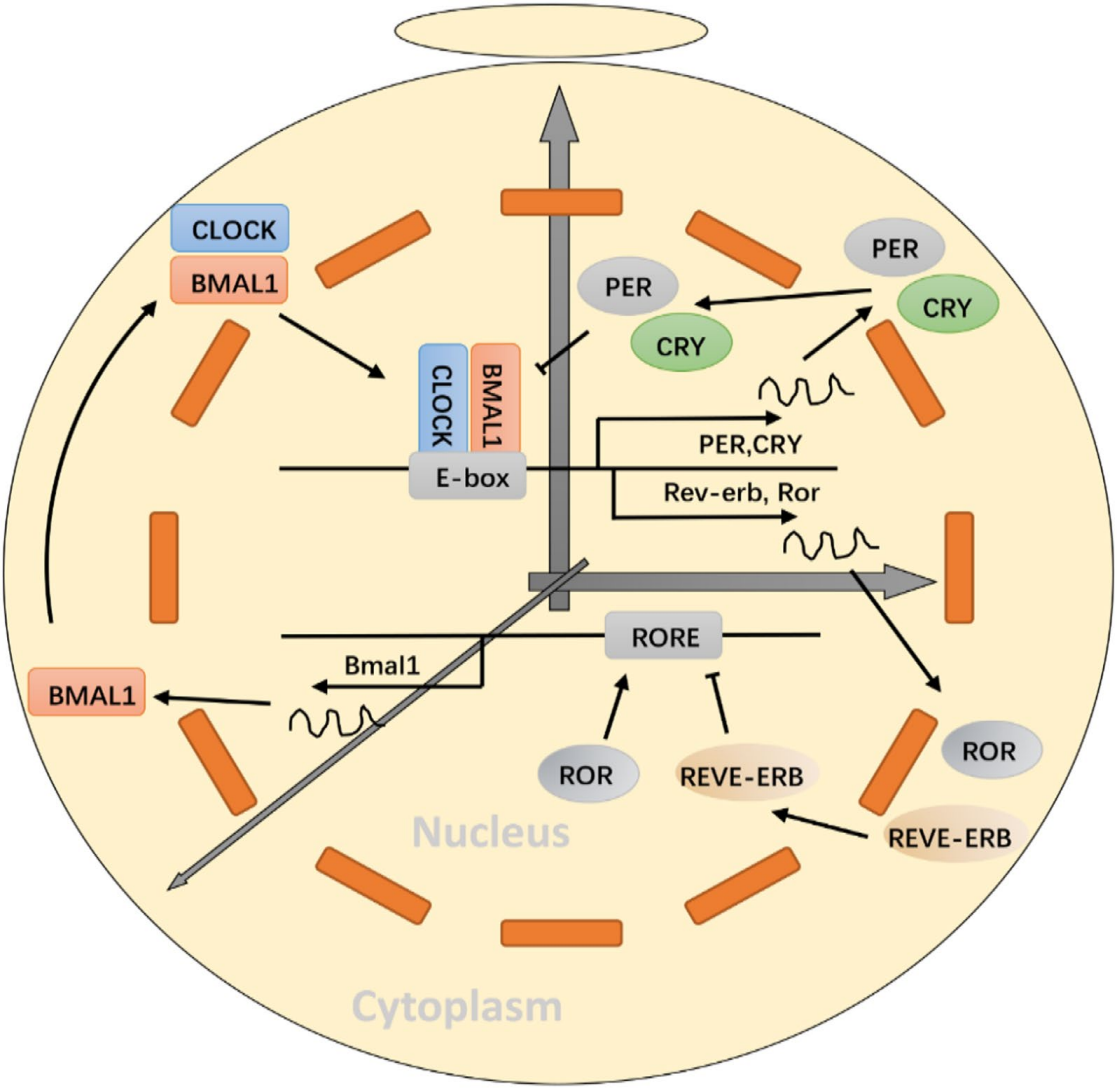
Schema of the circadian clock system—the transcription-translation feedback loops. The circadian clock consists of a network of TTFL that generates endogenous circadian rhythm. TTFL includes two loops: (1) The first loop of TTFL begins with BMAL1:CLOCK complex translocating into the nucleus, activating transcription of target genes containing E-box cis-regulatory enhancer sequences in their promoter regions, such as PER and CRY. The CRY and PER are then transferred to the nucleus and interact with CLOCK:BMAL1 complex to inhibit their own transcription. The decrease of PER and CRY protein levels reduces the suppression of BMAL1:CLOCK activity, which allows for the establishment of a new oscillatory cycle. (2) In the second loop, the REV-ERBα/β repressor and the RORα/β activator proteins co-maintain the periodic expression of BMAL1

Macroscopically, amplitude, phase, and period are key rhythmic parameters driven by the circadian system. Microscopically, circadian rhythm disorders can be limited to alterations in period due to defects in the core molecular mechanism, i.e., the TTFL [7]. TTFL is mainly regulated through heterodimeric partnership between the brain and muscle aryl hydrocarbon receptor nuclear translocator (ARNT)-like 1 (BMAL1) and the circadian locomotor output cycles kaput (CLOCK) (Fig. 2). Period (PER) and cryptochrome (CRY) are involved in this pathway. In addition, the TTFL is complemented by a second loop, in which the REV-ERBα/β repressor and ROR (retinoic acid receptor-related orphan receptor) α/β activator proteins co-maintain the periodic expression of BMAL1 [8, 9] (Fig. 2). The cycle of TTFL is about 24 h [10, 11].

Several studies have reported the occurrence of circadian disruption in PD or AD animal models, including changes in circadian rhythm, sleep pattern or clock genes in model animals including mice, rats, *Drosophila* and zebrafish. The current reports on dysrhythmia in PD and AD models, including neurotoxin-induced and transgenic animal models, are summarized in Tables 2 and 3.

**Table 2:** Circadian rhythm disorder in PD models

Species	Genotype	Toxin	Results/effects	Reference
Mouse	MitoPark		Loss of dopamine leads to circadian alterations of the rest/activity cycle.	[272]
Mouse	A53T		Impaired light entrainment of the circadian system	[273]
Mouse	ASO		Diurnal and circadian rhythms of wheel running behavior are disrupted	[274]
Mouse		MPTP	Expression of Bmal1, Per1, Per2, Cry1, Dec1 and Rev-erbα shows decreased amplitude of circadian oscillation	[275]
Rat		6-OHDA	Endogenous circadian rhythm in constant darkness is disrupted.	[276]
Rat		6-OHDA	The mRNA levels of *Bmal1*, *Per2*, and *Clock* are decreased.	[277]
Rat		LPS or rotenone	The mRNA levels of *Bmal1*, *Clock*, *Npas2*, *Per1* and *Per2* are decreased.	[278]
Rat		6-OHDA	Rats housed in constant darkness are less active.	[279]
Rat		6-OHDA	The length of the locomotor activity period is decreased during the dark, and increased during the light period.	[280]
Rat		6-OHDA	Decreased amplitude of heart rate and heart rhythm	[281]
Rat		6-OHDA	The circadian rhythms of blood pressure and temperature are disrupted.	[282]
Monkey		MPTP	Loss of circadian locomotor activity in the absence of light/dark cues	[283]
*Drosophila*	pink1 and parkin mutants		The fragmentation of sleep, the anticipation of dawn	[12]
*Drosophila*	pink1 and parkin mutants		Weakened circadian rhythms in locomotor activity	[284]
*Drosophila*	TP-αS		TP-αS expression in neurons interferes with the circadian rhythm of aging flies	[285]
Zebrafish		MPP +	Decreased activity, sleep disruptions, and impaired habituation to repetitive startles	[286]

ASO: alpha-synuclein overexpressing; TP-αS: three alanine replacements by prolines (at positions A30P, A56P and A76P)

**Table 3:** Circadian rhythm disorder in AD models

Species	Genotype	Toxin	Results/effects	Reference
Mouse	3×Tg		Decreased nocturnal activity, increased daytime activity, and shorter free running time	[287]
Mouse	TgCRND8		More stereotypic behavior with increasing age	[288]
Mouse	APP^swe^/PS1^ΔE9^	Chronic sleep deprivation	Abnormal expression of Bmal1, Clock, and Cry1	[105]
Mouse	P301S tau		Perturbed oscillations in BMAL1expression	[289]
Mouse	Bmal1 KO		Disruption of daily hippocampal interstitial fluid Aβ oscillations and accelerated amyloid plaque accumulation	[164]
Mouse	Tg4510		Per2 and Bmal1 are evidently disrupted in the hippocampus	[290]
Mouse		Aβ31-35	Disrupted daily sleep‐wake cycle and circadian oscillation of *Bmal1* mRNA and *Per2* mRNA	[291]
Mouse		Aβ1–42	Rhythm absence under LD or DD conditions	[292]
Mouse	J20		Altered peak acrophase	[293]
Mouse	APP/PS1		Dysregulation of *Bmal1* mRNA and *Per2* mRNA	[294]
Mouse		Beta/A4 amyloid	Disruption of circadian regulation	[295]
Mouse	APP/PS1		Phase delays of ~ 2 h in the onset of daytime wakefulness bouts and peak wakefulness	[296]
Mouse	CRND8/E4		Intermediate disruptions in circadian rhythms	[297]
Mouse	5×FAD		Altered circadian behavior, and altered expression of Bmal1 and Per2	[298]
Mouse		Aβ31-35	Disturbances in circadian rhythms	[299]
Mouse		Aβ31-35	Altered expression of Per1 and Per2 in the SCN, hippocampus and heart	[300]
Mouse	Fus1 KO		More sleep time during the diurnal cycle	[301]
Mouse	APPSwe/PS1dE9		Alteration of levels and patterns in circadian rhythm of locomotor activity, and altered expression of Cry1 and Cry2	[302]
Mouse	3×Tg		Increased activities in the resting phase, decreased and scattered activities in the active phase, decreased overall activity intensities, amplitude, robustness, and increased intradaily variability; phase delay in the expression of *Per*1 and *Per2* mRNA in the SCN	[303]
Mouse		Aβ31–35	An unclear movement phase and resting phase and a prolonged free running period	[304]
Mouse	APP/PS1		A mild but persistent phase delay of nocturnal activity onset in LD conditions	[305]
*Drosophila*	Express human Aβ		Fragmentation of daytime sleep	[306]
*Drosophila*	Tau-deficient		Dysregulation of daily circadian rhythms and sleep patterns	[307]

3×Tg: three mutations that have been associated with AD in humans (APP LysMet670–671AspLeu, MAPT Pro301Leu and PSEN1 Met146Val); J20: Two familial AD mutations (two in human APP: Swedish (K670N/M671L) and the Indiana (V717F)); 5×FAD: Five familial AD mutations (three in human APP: K670N/M671L (Swedish),I716V (Florida),V717I (London) and two in human PSEN1: M146L,L286V)

### Sleep-wake activity

Sleep-wake activity is regulated by internally driven rhythm of the circadian clock. Disruption of the sleep-wake cycle has been found in a variety of PD and AD models with multiple assessment methods, including running wheels, infrared beams, piezoelectric systems, and electrophysiological measures such as EEG and EMG. Sleep-wake disturbances in PD and AD seem to be associated with genetic mutations. For instance, *Drosophila* with *pink1 (*phosphatase and tensin homolog-induced putative kinase 1) and *parkin* mutants show fragmentation of sleep [12]. Heterozygous (D409V/WT) GBA1 (glucocerebrosidase 1) mutant mice show increased NREM sleep and reduced REM sleep durations [13]. Additionally, α-synuclein BAC transgenic mice exhibit REM sleep without atonia (RSWA), which is a key feature of REM sleep behavior disorder (RBD). Regarding the sleep alterations in animal models of PD and AD, Fifel, Medeiros and their colleagues have comprehensively discussed this topic, together with the strengths and limitations [14, 15].

The sleep-wake activity may be related to proteostasis in the brains of patients with neurodegenerative diseases. A few studies have shown that the sleep-wake activity regulates the clearance of misfolded proteins in the glymphatic system of the brain [7]. Interrupted sleep has also been shown to increase the level of Aβ protein in the cerebrospinal fluid [16, 17]. One possible explanation is that Aβ or α-synuclein induces sleep disorders, which in turn hinders their clearance by the lymphatic system, and ultimately accelerates the pathological progression of the disease.

## PD

Recent attempts to redefine PD have viewed it as a complex combination of motor and non-motor disorders, with a natural history that includes a prodromal phase dominated by a range of non-motor symptoms (e.g., sleep, psychiatric disorders, autonomic dysfunction, cognitive impairment and sensory deficits) [18]. As with parkinsonian motor abnormalities, non-motor symptoms may also exhibit diurnal fluctuations that change throughout the course of the disease. A dysfunctional circadian system is therefore expected to exacerbate the clinical symptoms of PD patients. It is reasonable to link the symptom fluctuations in PD patients with dysregulation of circadian rhythms affected by chronobiology [19].

**Fig. 3:**
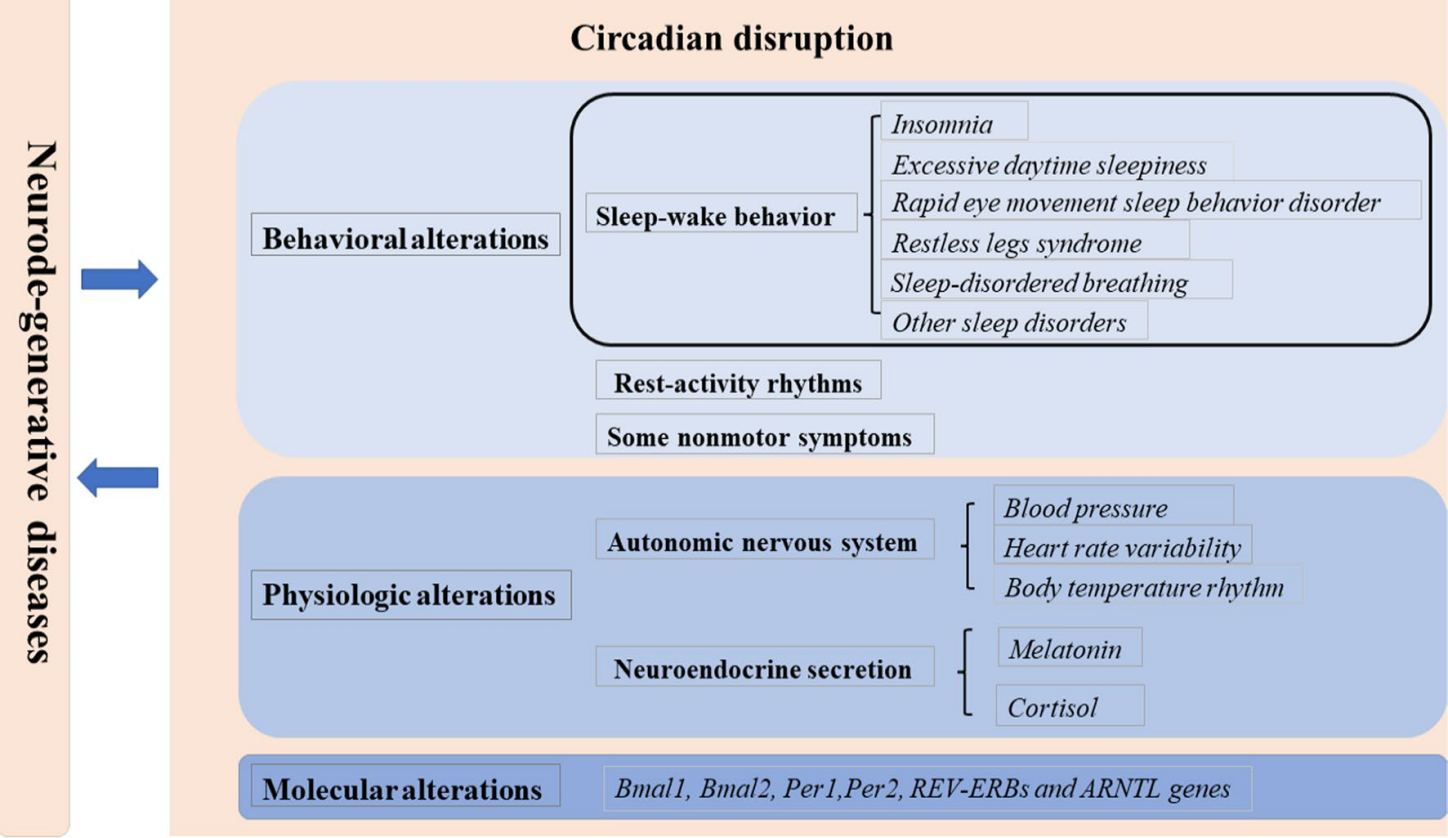
Circadian dysfunctions in neurodegeneration. Circadian dysfunctions in neurodegeneration can be classified into three categories: behavioral, physiologic, and molecular alterations. There are bi-directional relationships of circadian rhythm and sleep disorder with neurodegeneration

Diurnal oscillations are present in characteristic motor and non-motor symptoms of PD. Circadian biomarkers such as melatonin and clock gene, and the neurological processes underlying circadian rhythm, are altered by not only the neurodegeneration of PD, but also dopaminergic treatments used to mitigate parkinsonian symptoms [20]. In general, these circadian dysfunctions in PD can be classified into three categories: behavioral, physiologic, and molecular alterations (Fig. 3).

### Behavioral alterations in PD

The rest-activity rhythm is often characterized using nonparametric analysis of actigraphy data. The circadian rhythm disruption in PD is characterized by a reduction in the amplitude of the circadian rhythm, resulting in an overall flattening of the rhythm, but with no significant shift in circadian phases [4, 21]. One recent study quantified the actigraphy data, and found that the activity rhythms are associated with disease severity and fluctuations of symptom intensity [22].

Non-motor symptoms in PD are associated to a certain extent with an impaired circadian rhythm. Increasing lines of evidence have shown that circadian dysfunction plays a role in cognitive impairment associated with PD, either by directly affecting cognition or indirectly by exerting effects on sleep and alertness [23]. Several other functions (emotional disorders and gastrointestinal dysfunctions) that are relevant for optimal behavior are known to be altered in PD. However, their link with the circadian clock remains poorly understood [4].

Sleep disorders are the most common non-motor symptoms and comprise the entire spectrum of sleep disorders, mainly presenting with disorders of regulation of sleep and wakefulness (such as insomnia and daytime sleepiness), parasomnias (mainly REM sleep behavior disorders, but also, albeit more rarely, sleepwalking and overlap parasomnia), sleep-related movement disorders (restless leg syndrome, RLS) and sleep‑disordered breathing (SDB) [24]. Insomnia is a frequent symptom in PD and most PD patients often complain about sleep fragmentation and early awakening. The prevalence of insomnia in PD increases over time and requires periodic assessments. PD patients with depressive symptoms, motor fluctuations and the use of higher doses of dopamine agonists tend to suffer more severe insomnia [25]. Interestingly, insomnia is often one of the characteristics of PD where motor symptoms are improved upon awakening from sleep and prior to drug intake. However, the underlying mechanism of this phenomenon (“sleep benefit”) is unclear, and there is no direct evidence for its correlation with the circadian type predominance [26]. Excessive daytime sleepiness (EDS) is a major health hazard in PD, affecting 21%–76% of PD patients with an incidence of 6% per year [27]. In our previous study employing 586 PD patients, male sex, disease duration and depression were found to be main risk factors for EDS in PD patients, while depression was a predictive factor for poor night-time sleep quality in all PD patients, whether they were male or female, and had early- or late-onset PD [28]. Circadian dysfunction may underlie the excessive sleepiness in PD. Compared with PD patients without EDS, patients with EDS have significantly lower amplitude of the melatonin rhythm and 24-h area-under-the-curve for circulating melatonin level [29]. RBD is a parasomnia, characterized by dream-enacting behaviors (DEB) and nightmares linked to RSWA. Compared with PD without RBD, PD with RBD exhibits clinical heterogeneity of motor and non-motor symptoms. More specifically, both DEB and RSWA are associated with the severity of PD. DEB symptom might fluctuate or disappear over time whereas RSWA may continue to develop as PD progresses [30]. RLS has one essential diagnostic characteristic, the presence of circadian variation of symptoms. The circadian clock-controlled gene, *Tef*, is also associated with sleep disturbances in PD, including RLS symptoms [31]. SDB is not more frequent in PD than in the general population. We previously explored the clinical characteristics of PD with comorbid obstructive sleep apnea (OSA), and found that age and male gender are risk factors for OSA in PD [32]. OSA may exacerbate neurodegenerative processes in PD. It is hypothesized that the OSA-related intermittent hypoxemia leads to oxidative stress, neuroinflammation, cerebrovascular effects, disruption of the glymphatic function by sleep fragmentation, and changes in the integrity of the blood–brain barrier  [33].

Nocturia is a condition that is  caused by the dampening or reversal of the daily pattern of urine excretion, which is associated with poor quality of life, falls, and institutionalization in PD. The prevalence of nocturia ranges between 76% and 86% according to a previous questionnaire survey [34]. Recent advances in circadian biology and sleep science have raised the importance of considering nocturia as a form of circadian dysfunction, with a focus on the influence of circadian genes on the bladder as studies have demonstrated circadian gene cycling in all levels of bladder tissue, and abnormal nocturnal urine production [35].

### Physiologic alterations in PD

The physiological facets of circadian dysfunctions in PD include dysfunction of the autonomic nervous system and dysrhythmias of neuroendocrine secretion.

Rhythmic abnormalities in the autonomic nerve function in PD are well recognized, including reversal or even a full arrhythmia of blood pressure and heart rate variability (HRV), and impairment of the core body temperature (CBT) rhythm [20]. Nocturnal hypertension is nearly ubiquitous in PD, and most PD patients exhibit either a blunted nocturnal fall of blood pressure or higher blood pressure during the night than during the day (known as reverse dipping), leading to the difference between daytime and night-time BP closed [36]. HRV, an index for the autonomic, especially parasympathetic functions, is lower all day but higher at night in individuals with PD than in healthy controls [37]. Research has shown that impaired HRV may be related with the disease severity, motor symptom duration and dopaminergic dose in PD [38]. PD patients also show disruptions of circadian thermoregulation, with significant reductions in the mesor (the mean value around which the core temperature rhythm oscillates) of the CBT rhythm and dampened CBT rhythms, both of which are strongly correlated with REM sleep [39].

Melatonin synthesis and corticosteroid secretion are directly or indirectly regulated by SCN, and can be used as markers of the central clockwork to reflect the endogenous rhythmicity [40]. Two well-known studies have examined rhythms of melatonin in PD. One study showed a significantly diminished amplitude and amount of melatonin secretion in PD patients (*n* = 20) receiving stable dopaminergic therapy compared with controls (*n* = 15). Among PD patients, those with EDS exhibit the most prominent impairment in circadian melatonin secretion [29]. The other study also suggested reduced circulating melatonin levels in patients newly diagnosed with PD (*n* = 30), compared with the matched controls (*n* = 15) [41]. Neither of these studies found any difference in the timing of melatonin onset or offset [29, 41]. Different from these observations, some other studies [42] found an earlier peak of nocturnal melatonin level in PD patients receiving levodopa than in the control group. However, secretion and diurnal rhythmicity of some other circadian-modulated hormones are unaffected in PD, including growth hormone, thyroid stimulating hormone, prolactin, as well as certain fat tissue-associated hormones [43, 44].

## Circadian disruptions in AD and other neurodegenerative disorders

Individuals with AD often experience more severe circadian disruptions than the healthy elderly, which in turn exacerbate neurodegeneration in AD [45]. In the following, we will discuss in detail the behavioral and physiological circadian alterations in AD.

### Behavioral alterations in AD

#### Sleep disturbances

The prevalence of sleep disturbances in AD is roughly 14%–69% [46, 47]. Sleep-wake disorders, including insomnia and EDS, are the most common form of sleep disturbances in AD. Additionally, SDB and RLS are also frequently observed in AD patients. As for parasomnia, RBD is rarely reported in AD when compared to PD and dementia with Lewy bodies (DLB), while evidence for the presence of NREM parasomnia in AD is lacking.

A multicenter study reported that sleep-wake disorders occur in over 50% of AD patients [48] and are primarily manifested as increased sleep latency, nocturnal awakenings, excessive daytime naps, difficulty in maintaining sleep, and early awakening [47, 49]. Sleep-wake disorders could precede the development of classic AD symptoms and may progress throughout the course of the disease [50]. Long-term disruption of sleep could result in exacerbation of neuropsychiatric or behavioral symptoms around the timing of sunset in 2.5%–66% of AD patients [51–53], which is called sundowning, or sunset phenomenon. Studies suggest that the phase delay of the body temperature and the hormone secretion patterns may contribute to this unique phenomenon [54].

The occurrence of RLS in AD patients is around 4%–6% [55, 56]. In the context of PD, RLS is more frequently observed with a prevalence of 14% [57]. RLS is rarely reported in patients with multiple system atroph (MSA), progressive supranuclear palsy and HD [58–60]. The relatively high prevalence of RLS in PD may be attributed to the common dysfunction of the dopaminergic system as some researchers proposed [61]. However, controversies remain on this issue [61]. In fact, diagnosis of RLS in AD can be quite challenging due to the inability of patients to accurately report. Additionally, RLS and sundowning may have common symptoms (i.e., agitation) and timing (i.e., late afternoon), making it more difficult to achieve a reliable diagnosis. Therefore, Richard et al. have further proposed a new diagnostic method, which combines a novel behavior observation test with clinical measurements and comorbidities, and yields a relatively high accuracy [62].

SDB has been reported in 15%–54% of AD patients [56, 63, 64]. Emerging evidence suggests that SDB serves as an independent risk factor for the development of AD [65, 66]. Indeed, there seems to be a bidirectional relationship between SDB and neurodegeneration. A recent meta-analysis pooling several randomized controlled trials indicated that continuous positive airway pressure treatment for SDB in AD patients can ameliorate cognitive performance, mood, EDS, slow-wave sleep (SWS) and apnea–hypopnea index (AHI) [67]. Although studies with a larger sample size are warranted, these studies shed light on the possibility of reversing cognitive decline in AD patients with comorbid SDB.

Accumulating evidence has suggested that RBD should be considered as the prodromal stage of α-synucleinopathies [68]. However, apart from a report of a rare case of drug-induced RBD in AD [69], two cross-sectional studies reported that the prevalence of RBD is approximately 10% in AD [70–72] and several longitudinal studies revealed the development of AD in RBD patients [73–76]. Notably, the diagnosis of AD in these longitudinal studies was not totally confirmed by autopsy. In addition, DLB and AD could be difficult to differentiate due to some overlapping clinical manifestations when postmortem analysis is lacking. Nonetheless, even autopsy findings are obtained, there remains a possibility of misdiagnoses when different neuropathological techniques are applied. For instance, a 72-year-old male patient diagnosed as AD was later defined as ‘Lewy body variant of AD’ when a new staining method was used [77–79]. Therefore, whether the development of AD in RBD patients indicates a mixed subtype of dementia or it is merely a technically false diagnosis needs further observations.

As for NREM parasomnia, although AD patients experience a reduction of SWS and spindle activity [80], there is limited evidence for any definitive NREM parasomnia in AD patients.

#### Rest-activity rhythm changes in AD

The rest-activity rhythm is one of the most commonly studied indicators of circadian disruption in AD. Recent studies showed that AD patients exhibit increased fragmentation of rest-activity rhythm, increased night-time awakening, and decreased daytime activity. However, there are mixed findings regarding alterations of the circadian amplitude or phases [81, 82].

### Physiological alterations in AD

Physiological facets of circadian dysfunctions in AD mainly include dysfunctions of the autonomic nervous system and dysrhythmias of hormone secretion.

Dysregulations of the autonomic function in AD primarily manifest as orthostatic hypotension [83], non-dipping or reverse dipping in mainly systolic blood pressure [84], reduction in HRV index [85] and a phase delay in CBT [86]. Intriguingly, Kim et al. reported that HRV might be a potentially useful tool for early differentiation between AD and DLB [87].

Additionally, AD patients exhibit reductions of melatonin levels and a phase delay in melatonin secretion [88, 89]. Findings in HD are largely inconsistent with those in AD [90, 91]. As for cortisol, elevated cortisol levels and a phase-advanced cortisol-secretion pattern are observed in AD patients [92, 93], which bear some resemblance to those in late-stage HD [94]. In addition to higher cortisol levels, early-stage HD also displays an increased amplitude in the cortisol secretion rhythm [90].

### Pathogenic mechanisms linking circadian rhythms, sleep and AD

The mechanisms linking circadian rhythms, sleep and AD have not come to a definitive conclusion. Mounting evidence has suggested a bidirectional relationship between them. Previous studies indicated that circadian dysfunctions worsen neurodegeneration in AD through cholinergic disturbances and melatonin loss. Autopsy studies have revealed loss of neurons in the SCN of AD patients, particularly neurons expressing vasopressin, melatonin receptor type 1 and vasoactive intestinal peptide (VIP) [95, 96]. In addition, the decreased melatonin levels [97] are associated with the rest-activity rhythm disorder [95, 98]. Moreover, AD patients with circadian dysfunctions show loss of ipRGCs [82, 99], which was reported to be associated with Aβ deposition in one study [82]. Additionally, AD patients or mouse models with cholinergic disturbances demonstrate impaired circadian pattern [100, 101]. Melatonin suppresses Aβ generation and amyloid fibril formation. There is evidence showing a reduction of melatonin level in the prodromal and progressive stages of AD [102]. On the contrary, circadian disruptions promote neurodegeneration in AD. Sleep deprivation or disruption significantly increases neuroinflammation and subsequent Aβ production in the cerebrospinal fluid (CSF) of AD patients [103], increases Aβ and phosphorylated tau (ptau) levels in transgenic AD mouse models [104, 105], and decreases glymphatic flow and Aβ clearance in human CSF and in animal models [104, 106], which may lead to further progression of neurodegeneration in AD. Notably, chronic sleep disruption may increase the dissemination of tau protein in neural networks [107]. Musiek et al. have reported severe astrogliosis, oxidative injury and synaptic degeneration in Bmal1-deleted mice, indicating that circadian dysregulation of the neuronal redox homeostasis may also contribute to neurodegeneration in AD [1].

### Other neurodegenerative disorders

Circadian disruption and sleep disorders are also observed in other neurodegenerative disorders, including MSA, DLB, frontotemporal dementia (FTD), and HD.

Although no α-synuclein deposition is found in either the SCN or the pineal gland in MSA cases, the circadian dysfunction may be secondary to degeneration of other systems, such as the autonomic networks [108]. The circadian rhythm is regulated by VIP-expressing neurons, which are more involved in autonomic control and depleted in the SCN of patients with MSA [95]. MSA has a wider impairment of circadian regulation of endocrine and autonomic functions, such as plasma cortisol concentration [109], blood pressure, gastric myoelectrical activity [110], and nocturnal polyuria.

Patients with DLB present with decreased amplitude of CBT during night and more severe daytime sleepiness than controls, as well as more frequent RBD than AD and healthy controls [111]. Notably, inclusion of RBD has been proven to improve the diagnostic accuracy of DLB [112].

Although neurodegenerative disorders exhibit fragmentation of sleep, patients with FTD [113] show phase-advanced activity rhythm while HD patients [114] are with phase delay.

## Bi-directional relationship of circadian rhythm dysregulation and sleep disorder with neurodegeneration

Although initially considered to occur consequently after disease onset, the impairment of sleep and circadian rhythm is now recognized to predate clinical diagnosis or occur in the early stage of neurodegeneration [4, 115, 116], posing individuals at risk of the incidence or progression of neurodegenerative diseases. Therefore, a potential bi-directional relationship can be inferred between sleep disorder/circadian rhythm dysregulation and neurodegeneration.

### Sleep disorders accelerate neurodegeneration

#### RBD

RBD is well recognized as a strong prodromal predictor of α‑synucleinopathies, especially PD. Schenck et al. noted for the first time that 38% of individuals with initial diagnosis of isolated RBD (iRBD) finally developed a parkinsonian disorder within 3.7 years [117]. Now, it is recognized that most iRBD individuals will end up being diagnosed as α‑synucleinopathies, including PD, PD dementia, DLB and MSA. The estimated overall conversion rate is 6.3% per year and 73.5% within 12 years in a large-scale multicenter study [118]. For those with longstanding iRBD without overt conversion, crucial prodromal PD markers such as olfactory loss, constipation and mild parkinsonism are commonly observed in these populations [119]. This suggests that iRBD is consistently influenced by an underlying neurodegenerative process, which is also supported by findings of widespread Lewy body pathology and α-synuclein in iRBD individuals [120]. By pooling studies on biomarkers of iRBD–PD association, a recent meta-analysis showed that motor dysfunction, constipation, orthostatic hypotension, hyposmia, mild cognitive impairment, and abnormal color vision in iRBD are significantly associated with subsequent PD risk [121]. Other biomarkers on imaging, polysomnography (PSG) or EEG can also be used to monitor the neurodegenerative process in iRBD [122]. Considering the long interval between onset of iRBD and overt α‑synucleinopathy, close monitoring of these biomarkers may provide opportunity for use of disease-modifying treatments in the prodromal stage of neurodegeneration. Approximately 3–11% of RBD patients were recorded in previous studies to develop incident clinical AD [73, 74]. However, there is still limited evidence from longitudinal or prospective studies on the conversion of RBD to mild cognitive impairment (MCI) or AD. Compared to individuals without probable RBD (pRBD), those with pRBD have a 2.2-fold increased risk of MCI [123]. In another population-based study, individuals with pRBD who eventually developed MCI and subsequent dementia were actually highly consistent with a diagnosis of DLB [124]. By further evaluating previous neuropathological studies with 16-year follow-up [117], researcher found that the clinically identified AD patients developing from RBD exhibited a ‘mixed pathology’ but not ‘pure AD’, that is, they had histopathological features of both AD and DLB. In sum, the clinically observed RBD–AD association may be due to the presence of dementia in α‑synucleinopathies.

#### Insomnia

Chronic insufficient sleep plays a vital role in the pathological process of AD pathology. Both animal and human studies showed that sleep deprivation causes increased Aβ formation and deposition [103, 104] and contributes to neurodegenerative  process that affects neuroinflammation and synaptic homoeostasis [125]. Besides, objective short sleep duration and circadian rhythm disruption may exert an add-on effect on the risk of AD. Xu et al. [126] systematically reviewed studies on the association of sleep with all-cause cognitive decline or dementia, and found that insomnia significantly contributes to an increased risk of incident AD but not vascular dementia with a pooled relative risk of ~ 1.5. In a subsequent longitudinal analysis of U.S. adults aged over 65 years, individuals with an increase in the severity of insomnia over time have 41%–58% higher risk of memory decline or dementia [127], suggesting the importance of early sleep health for AD prevention. In contrast, studies on the impact of insomnia on the susceptibility to PD are limited. In a large registry-based case–control study, significantly higher incidence of insomnia (RR = 1.38, 95% CI 1.11–1.70) was observed 2 years before diagnosis of PD [128]. A recent study differentiating insomnia subtypes found that sleep-onset insomnia, in comparison to maintenance insomnia, is associated with more motor, cognitive, and autonomic symptoms [129]. To be noted, insomnia is not persistent throughout the disease course [25] and their subtypes [130] may change in PD. In addition, previous studies revealed that the observed insomnia–PD association may disappear with longer follow-up duration [128, 131]. Also, some studies showed that present insomnia could not predict conversion to neurodegeneration in iRBD individuals [132, 133]. Therefore, it is likely that insomnia is more likely to be a prodromal symptom instead of an etiology of PD.

#### OSA

OSA-related intermittent hypoxia, neuroinflammation and sleep fragmentation have been proven to accelerate neurodegeneration by disturbing Aβ clearance and aggravating neurofibrillary tangles of tau in AD [134], or by damaging the nigrostriatal dopaminergic system and promoting aggregation of α-synuclein in PD [135]. Mounting studies have confirmed the emerging role of SDB, either self-reported sleep apnea or PSG-proven OSA, in the incidence or early progression of neurodegenerative conditions [66, 135, 136]. For example, Yaffe et al. [136] found that women with OSA having AHI > 15 have a higher risk of developing MCI or dementia, with an odds ratio of 1.85. This was further confirmed in a recent prospective cohort with large sample size, which showed that only severe OSA individuals with AHI > 30 have 66%–135% higher risk of developing all-cause dementia or AD [137]. More evidence from the AD biomarker perspective suggests that cognitively intact OSA individuals have higher Aβ burden indicated by blood, CSF and imaging biomarkers compared to individuals free of OSA [138, 139]. Moreover, adherence to positive airway pressure therapy may lower the odds of incident diagnosis of AD or MCI and slow cognitive impairment or its progression to AD [140, 141]. Consistently, another longitudinal analysis revealed increased risk of PD in individuals with sleep apnea [142, 143]. Subsequent meta-analysis confirmed the association of OSA with incident diagnosis of PD by pooling 12 eligible studies and revealed similar risk between males and females [144]. These findings are further supported by Sun et al. showing that levels of plasma total and phosphorylated α-synuclein are significantly elevated in OSA individuals and are inversely correlated with oxyhaemoglobin saturation [145], indicating that OSA-related hypoxia is involved in the pathogenesis of PD pathology. Taking AD and PD together, however, a recent mendelian randomization study failed to reveal a causal association between genetically-predicted OSA and risk of AD or PD [146]. An explanation is that the OSA–neurodegeneration association may be not unidirectional but bi-directional, and that confounding factors such as comorbidities may also play a role [146].

#### EDS

EDS is another emerging predictor of neurodegeneration. Many studies have identified the temporal association between EDS and risk of dementia or AD, which is independent of comorbid chronic diseases or conditions [147–149] but can be partly confounded by lack of daily physical activity or social engagement [149]. In revealing the sleepiness–AD pathologic association, an imaging study by Carvalho et al. reported that EDS patients with normal cognition show more prominent grey matter thinning in age-susceptible regions which  is usually observed in AD pathology [150]. In 283 dementia-free participants aged 70 years and over receiving Pittsburgh compound-B positron emission tomography, the same team found that the baseline EDS  is longitudinally associated with increased Aβ accumulation in the cingulate gyrus and precuneus regions, indicating that the elderly persons with EDS are more susceptible to AD pathologic alterations [151]. This is supported by similar sleep-related animal studies showing abnormal Aβ generation or Aβ deposition due to impaired glymphatic clearance [104] and dysregulated cortical slow-wave activity [152]. EDS also serves as a robust prodromal marker of PD. Two studies have indicated that daytime sleepiness leads to a  2–3-fold increased risk of incident PD among the general population [153, 154]. However, results were inconsistent on if EDS predicts conversion to neurodegeneration in iRBD individuals [74, 132, 133]. The inconsistency may be due to the differences in sample size as well as ethnic and clinical backgrounds of the participants. In a neuropathological study, Abbott et al. combined EDS assessments and α-synuclein staining in postmortem brain samples from 211 men to identify the relationship between Lewy body pathology (LP) and EDS [155]. They found that the prevalence of EDS became significantly increased only when Lewy body pathology extensively infiltrated into the neocortex (equivalent to Braak stages 5 and 6). However, absence of Lewy body pathology was noticed in 41% cases of EDS at the time of autopsy. In other words, it was uncertain whether EDS preceded Lewy body pathology in the remaining 59% cases, and whether sleep augmentation of the clearance of tau and α-synuclein underlies the relationship between sleepiness and PD pathology remains unknown.

#### RLS and other sleep disorders

In viewing the association between other sleep disorders and neurodegeneration, prospective cohort studies found that RLS is associated with a 1.5–2.5-fold higher risk of incident PD. However, it remains unclear if the dopaminergic system is a pathologically link between RLS and PD due to the absence of evidence from basic research [156]. When examining the overall effect of sleep-related movement disorders (SRMD) on dementia, Lin et al. [157] found individuals with diagnosis of SRMD had a 3.9-fold higher risk of incident all-cause dementia and the observed association was more prominent in women and in those aged 45 to 64 years. For NREM parasomnia, a recent large-scale cross-sectional study including 25,694 men showed that sleepwalking was associated with 4.8-fold odds of having PD, regardless of the confounding factors [158].

### Circadian disruption aggregates process of neurodegeneration

Circadian activity disturbance, initially considered as symptoms of neurodegeneration, is believed to be involved in the occurrence or progression of neurodegenerative process. In other words, circadian disruption may precede clinical symptomology or add to the risk of neurodegenerative diseases.

Only few studies have examined the temporal association between circadian rhythm disruption and PD [159–161]. In a prospective study including 2930 men, Len et al. found that those who self-reported napping time of more than 1 h/day had a twofold risk of developing PD, compared to those with no EDS or having napping < 1 h/day [159]. From the same cohort, they also reported that weakened circadian rhythmicity was associated with increased risk of incident PD and such association remained significant even after excluding PD diagnosis in the first 2 years of follow-up [160]. These findings indicated that circadian rhythm disruption could be a prodromal marker for PD. To minimize the effect of reverse causality and potential confounding factors, recent mendelian randomization study using UK Biobank from European ancestry found that morning chronotype had an inverse causal effect while M10 (average activity during the most active 10 consecutive hours of the day) had positive causal effect on the later onset age of PD [161]. They also noticed that better sleep efficiency was causally associated with a decreased AD risk. However, temporal associations between circadian rhythm and PD or other neurodegenerative diseases such as HD and amyotrophic lateral sclerosis have not been thoroughly investigated as in AD and dementia. In addition, behavioral indicators like actigraphy do not necessarily parallel endogenous circadian biomarkers which maintain 24-h oscillations even in the case of sleep disturbance. Therefore, comprehensive evaluations of both behavioral and biological markers of circadian rhythm are needed to provide more convincing and detailed evidence on circadian rhythm disruption in PD-related neurodegeneration.

In a case–control study, more evident circadian misalignment, assessed by PSG and dim light melatonin onset, was noticed in MCI patients before AD diagnosis [162]. A recent study by Musiek et al. including 189 healthy elders showed that those with preclinical AD, as assessed by PET, demonstrated increased rest-activity rhythm fragmentation independent of aging and sex [163]. This finding suggested that circadian disruption is involved early in AD pathogenesis. In line with clinical findings, research on animal models revealed disruption of daily Aβ oscillations in the hippocampal interstitial fluid and acceleration of amyloid plaque accumulation in mice with disturbed circadian rhythm [164]. Inhibition of the circadian repressor REV-ERB is also associated with enhanced transcription of core clock gene *BMAL1* and increased Aβ clearance [165]. These observations add to the evidence of the potential role of circadian regulation in AD.

The temporal association between circadian rhythm and AD or dementia has been explored in many epidemiological studies [166–172]. Habits of dysregulated circadian rhythm, such as long-time night-shift or delayed rising time, are associated with increased risk of dementia among healthy populations [166, 167]. Assessment of behavioral indicators of circadian rhythm disruption via actigraphy can more objectively reveal the association [173]. Based on the same cohort of Study of Osteoporotic Fractures including 1283 women, Tranah et al. [174] and Walsh et al. [168] both found an association of decreased circadian amplitude with higher risk of cognitive decline or dementia during the 5-year follow-up. Besides, they reported worse cognitive decline in association with phase delay, different from the report by Rogers-Soeder et al. [169], which showed phase-advanced acrophase in the men-only cohort. In a prospective cohort study including 1401 healthy older adults, lower amplitude of 24-h activity rhythm and higher intradaily variability for hourly fragmentation of activity rhythm are associated with higher risk of developing AD dementia, while lower interdaily stability of 24-h activity rhythm predicts higher risk of transition from MCI to AD dementia [170]. The observed parameters also worsen as dementia deteriorates, indicating that the relationship between circadian disruption and AD progression is bidirectional. To specify the affected aspects of circadian rhythm, novel or refined analysis  has been applied in some studies [171, 172]. A 24-h time-limited difference of circadian rhythm was observed in individuals with preclinical PD in various periods in comparison to healthy controls [172]. By using parametric and nonparametric analysis, reduced overall rhythmicity, lower amplitude and activity level, and later activity timing were revealed to be associated with development of MCI and dementia [175]. However, there are also some studies showing no association of 24-h activity rhythm fragmentation with dementia risk or preclinical AD [172]. Discrepancies in these findings could be due to different sample sizes, study populations or statistical methods.

### Progression of neurodegenerative disease promotes circadian disruption and sleep disorders

Although sleep-wake disturbance is commonly found in PD, the rhythmic change throughout disease process has been rarely noticed. Using continuous actigraphy, a study showed that PD patients with higher Hoehn and Yahr stage are significantly more active later in the day [22], indicating an alteration of circadian rhythm in relation to disease severity. Additionally, the coexistent sleep disorders in PD might increase the variability of circadian rhythm. In a study of 15 iRBD patients, 31 PD patients and 6 DLB patients [111], Raupach et al. observed an inverse correlation between the CBT amplitude and RBD severity. Interestingly, the alterations in CBT were absent in PD patients free of RBD. This suggests that certain circadian rhythms might be particularly linked to RBD pathology and a further exploration of prodromal sleep disorders in PD is necessary. When discussing the longitudinal contribution of PD progression in relation to sleep disorders or circadian changes, the use of anti-PD medications is also of great importance [4]. In other words, whether these alterations result from dopaminergic treatment or from PD deterioration itself should be clarified. For example, a previous study found that only PD patients with levodopa-induced motor complications showed decreased ratio of melatonin secretion at night while such change was not observed in untreated patients or treated PD patients without motor complication [42]. Two other sleep disorders in PD, EDS and RLS, may also be consequences of dopaminergic therapy when symptoms aggregate as disease progresses. Drug-naive PD patients showed increased EDS severity from baseline to year 3 while no change was observed in the healthy control group. Meanwhile, the influence of dopaminergic medications on EDS was dose-dependent at years 2 and 3 of study follow-up [176]. The prevalence of RLS in untreated PD patients does not differ significantly from that of the general population, but is significantly increased in those receiving dopaminergic medication [177].

Across the disease continuum, studies using functional and biological indicators of circadian disruption in AD showed severer or irregular changes [88, 162, 178]. The severity of increased fragmentation of rest-activity cycle and overnight activity correlates with AD severity, with disturbances being most prominent in institutionalized patients [179]. The phase delay identified in AD patients is more prominent in those with advanced AD pathology [180]. As for core body temperature, a significant phase advance in circadian thermoregulation was observed in MCI patients compared to the healthy group [178] while phase delay was noticed in AD patients [180]. Similarly, for biological indicators such as melatonin level, MCI patients have a phase advance in melatonin secretion whereas those with mild-to-moderate AD exhibit a phase delay [88, 162]. These observations indicate that the neurodegenerative process can alter circadian rhythmicity and the latter can become more irregular as pathology deteriorates. Another question is that whether it is AD itself, but not aging, that promotes circadian disruption and sleep disorders. Initial view contends that individuals with AD have circadian alterations similar to those seen in healthy older adults, but with higher severity [181]. However, a recent study by Musiek et al. showed that preclinical amyloid pathology is associated with worse circadian fragmentation regardless of age [163]. This suggests that despite the same fragmentated circadian pattern, AD and normal aging drive circadian dysfunction in separate ways. Moreover, ageing alone is associated with a dramatically increased prevalence of preclinical AD to 30%–40% [182]. That is to say, in several epidemiological studies examining circadian dysregulation/sleep disorders preceding AD symptomology [168, 171, 175], the enrolled participants of advanced age might be already in the early stage of AD, which provides an interpretation for the observation that an underlying preclinical AD pathology may in turn lead to circadian and sleep dysfunction. The estimated prevalence of overall sleep disorders in individuals of AD is about 39% [46]. With self-reported assessment and overnight PSG, Hita-Yañez et al. found patients with MCI already showing disturbed sleep at both objective and subjective levels [183]. This work was further supported by a subsequent study showing significant relationship between Aβ deposition and sleep quality in preclinical AD [184]. As these cross-sectional observations were from an early stage of AD, this could be interpreted, from one perspective, that sleep is particularly sensitive to AD pathology. As dementia worsens, concurrent sleep disorders in AD such as EDS can be aggregated through potential mediating factors [184]. Comorbid depression and decreased social engagement resulting from impaired cognition in AD may add extra burden to EDS severity [185]. For SDB, around half of individuals with AD experience OSA and show five times higher odds of having OSA compared to age-matched cognitively intact individuals [184]. However, the longitudinal association between OSA or other specific sleep disorders (e.g., RLS) and AD progression has not been well explored to date [186].

As studies focusing on alterations of circadian rhythm/sleep disorder after diagnosis of neurodegenerative diseases are generally lacking, more studies are needed to longitudinally examine alterations of circadian rhythm and sleep disorders with progression of neurodegenerative diseases.

## Therapeutic strategies for circadian dysfunction and sleep disorders

### Non-pharmacological approaches

**Fig. 4:**
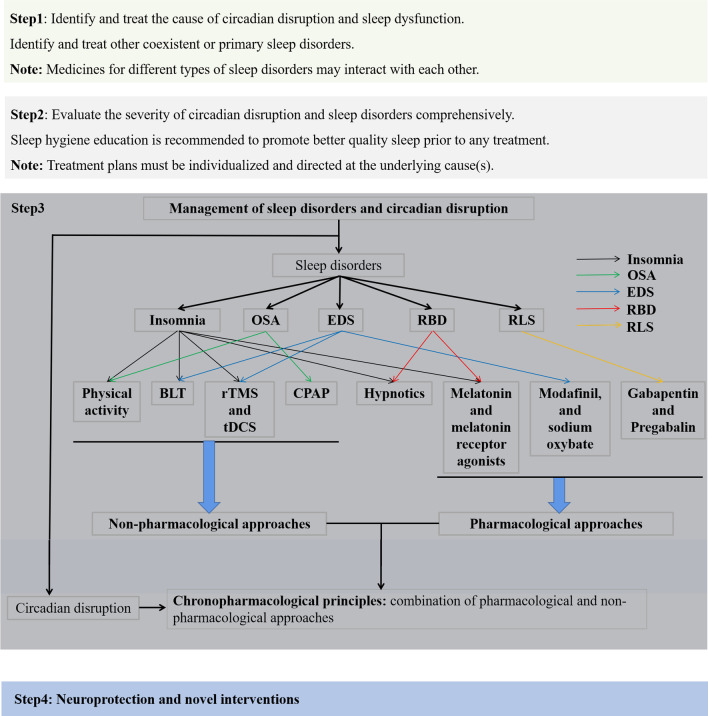
Management of circadian disruption and sleep disorders in patients with neurodegenerative diseases in four steps. About circadian disruption, chronopharmacological methods which combine both pharmacological and non-pharmacological approaches can be considered

Non-pharmacological approaches are considered as the first-line therapies for the management of circadian dysfunction and sleep disorders in neurodegenerative diseases. Treatment plans must be tailored at individual level. Prior to treatment, a comprehensive set of clinical, neuropsychological, neuroimaging, and electrophysiological assessments should be conducted, and the circadian disruption as well as the causes and subtypes of sleep disorders need to be carefully evaluated. Notably, sleep hygiene is recommended as an important behavioral and environmental practice in every treatment plan to promote better-quality sleep in PD [187] and AD [188] patients. As different medications for different types of sleep disorder may interact with each other, it is necessary to identify and treat coexisting or primary sleep disorders before making medication plans (Fig. 4).

#### Physical activity

Exercise is a circadian modulator. In PD, exercise can improve subjective sleep quality and objective PSG parameters. A retrospective study reported that intense physical and multidisciplinary exercise for 28 days improves the total PDSS score in PD patients [189]. Recently, Amara et al. [190] conducted the first study to demonstrate the impact of high-intensity exercise on objective sleep outcomes in PD patients and found that exercise  is more effective than sleep hygiene education in improving PSG parameters such as the total sleep time and sleep efficiency. Specifically, recent research suggested traditional Chinese exercise including Tai Chi [191], Baduanjin [192] and Qigong [193] as useful tools to improve PD sleep.

Similarly, walking for 30 min per day reduces the awake time by 33.1 min per night in AD patients [194]. Although one study has found that exercise could improve the daily cortisol rhythm in AD patients [195], the underlying mechanisms remain to be clarified. Adaptive neuroplasticity, which is beneficial for neuron re-organisation, is a suggested mechanism of the effects of physical activity [196].

When applying exercise in patients with neurodegenerative disorders, factors that may cause potential bias, including intensity, modality and compliance, need to be well controlled. A recent meta-analysis pointed out that moderate-to-maximal intensities rather than mild-to-moderate intensities of exercise have significant effects on subjective sleep quality [197]. Multi-modal exercise therapy at vigorous intensities is recommended [197].

#### Bright light therapy (BLT)

Light exposure is a powerful modulator of circadian rhythm and plays an important role in enhancing the rest-activity rhythm and thus promoting sleep in healthy individuals. Although the mechanisms of light therapy have not been well defined, retina, a key route of light entry into the brain, has been receiving more and more attention with regard to neurodegenerative diseases. Deposition of α-synuclein in retina has been well found in PD patients [198]. Light therapy could stimulate dopamine release by stimulating cells within the retina, thus improving the abnormal circadian rhythm and the motor symptoms of PD patients [199].

Recent studies found that BLT treatment is significantly associated with improvements of circadian rhythm and thus sleep quality of PD patients [200, 201], especially the subjective sleep quality and actigraphic measures, including sleep fragmentation and daily physical activity. In AD, a large double-blind, randomized, placebo-controlled trial found that light therapy (2500 lx) for 2 months could improve sleep quality and restore diurnal activity rhythms as measured by actigraphy [194].

BLT appears to be a feasible treatment for ameliorating sleep disorders in patients with neurodegenerative diseases. Although most studies recommend BLT as an alternative non-pharmacological method with few adverse effects such as headache [200, 202], the conclusion still needs to be confirmed in larger populations. Meanwhile, the effects of BLT may be confounded by medication, lifestyle, severity of disease, compliance of patients and co-morbidities [202].

#### Repetitive transcranial magnetic stimulation (rTMS) and transcranial direct current stimulation (tDCS)

rTMS and tDCS are two noninvasive brain stimulation techniques that can improve sleep quality in the healthy elderly.

In PD patients, rTMS therapy improves sleep fragmentation and sleep efficiency and reduces the average duration of nocturnal awakenings based on actigraphic results [203] and sleep scales [204]. Recently, rTMS has been demonstrated to improve daytime sleepiness in PD patients [205]. In AD patients, rTMS for 4 weeks significantly improves Pittsburgh Sleep Quality Index scores [206]. So far, only two studies have assessed the therapeutic efficacy on motor symptoms in HD and results are contradictory [207, 208]. tDCS enhances the slow-wave sleep [209], which is thought to play an important role in clearing Aβ during sleep.

### Pharmacological approaches

#### Melatonin and melatonin receptor agonists

Melatonin plays a role in regulating the circadian rhythm and promoting sleep. Replacement therapy with exogenous melatonin may have positive effects against sleep disturbances and even pathological progression of neurodegeneration. Several clinical studies have demonstrated the positive effects of melatonin on insomnia, RBD and rest-activity disruption in neurodegenerative disorders. However, the results are mixed. Melatonin could significantly improve subjective sleep quality and total sleep time in PD [210]. However, the effect of melatonin on EDS remains unclear. Of note, melatonin is recommended as the first-line therapy because it reduces RBD-related injuries with fewer side effects than clonazepam [211]. In the context of AD, one recent systematic review showed that melatonin shortens the sleep onset latency and increases sleep duration [212]. Several studies also proved that prolonged-release melatonin [213] and melatonin receptor agonists [214] can improve subjective sleep quality in both PD and AD patients. Although plasma concentrations of melatonin are shown to be reduced in HD [91], the efficiency of melatonin in HD patients has not been systematically investigated. Recently, a study demonstrated beneficial effects of melatonin in restoring clock gene expression in *Drosophila* model of HD, suggesting a promising clinical use in the future [215].

However, most melatonin-related studies have similar limitations. (1) The melatonin dose varied widely across studies (mostly 3–5 mg). (2) Most data were derived from case reports and long-term longitudinal studies are lacking. So far, most studies focused on the hypnotic effect rather than effects on the circadian rhythm. More studies evaluating alterations of the biomarkers of circadian rhythm during melatonin treatment are needed. (3) The circadian rhythm of melatonin secretion profile may be different among individuals, and can be influenced by other factors such as food, physical exercise and light. Solutions to these problems can increase the melatonin efficiency in personalized treatments.

#### Hypnotics

Hypnotics including benzodiazepines, non-benzodiazepines drugs, sedative antipsychotics, and sedating antidepressants, are widely used to treat insomnia in healthy adults. Recently, several studies have found its use for various sleep disorders in neurodegenerative disorders.

Clonazepam, a long-lasting benzodiazepine, is the first-line pharmacological option for RBD [216]. Shin et al. showed that clonazepam improves pRBD symptoms in patients with PD [217]. A 6-week randomized controlled trial showed that eszoplicone significantly reduces the number of awakenings after sleep onset and improves subjective sleep quality in PD patients [218]. Similar results have been obtained in AD patients [219]. In fact, current evidence for clonazepam is mainly based on case reports and observational studies [220, 221], thus more clinical trials are needed. Sedative antidepressants drugs like trazodone and doxepin are also proven to be effective for nighttime percent sleep in AD [222] and insomnia in PD patients [223].

It is important to note that hypnotics may also produce adverse effects such as memory deterioration and worsening of daytime sleepiness or sleep-related breathing disorders, especially in elderly adults [24]. Neurologists should well assess the risk/benefit profile before prescribing these hypnotics agents for patients with neurodegenerative diseases.

### Chronopharmacological principles: combination of pharmacological and non-pharmacological approaches

Chronotherapy is a therapeutic approach that incorporates an individual's circadian rhythm into disease treatment. It is based on the principle of prescribing drugs according to the different characteristics of an individual's circadian rhythm or combining both pharmacological and non-pharmacological approaches. Characterized by maximizing drug effectiveness and minimizing its side effects, chronotherapy was initially used mainly in the treatment of hypertension and in oncology. Recent studies have found that chronotherapy has promising applications in neurodegenerative diseases [194, 224, 225].

A study suggests that combining BLT with melatonin in demented patients may increase sleep efficiency, attenuate agitated behavior and even improve nocturnal restlessness for 3.5 years [224]. Dowling et al. found that 1 h of morning light exposure (2500 lx) for 10 weeks together with 5-mg melatonin in the evening significantly increases the daytime awake time and activity levels and strengthens the rest-activity rhythm of AD patients, compared to the light therapy alone [225]. MuCurry et al. found that combination treatment (walking, light, and sleep education) and each treatment alone have similar effects in improving sleep outcomes in AD patients [194]. Preliminary studies of chronotherapy in neurodegenerative diseases have shown promising findings. Personalized treatment plans are essential for effective implementation of chronotherapy, as the melatonin secretion curve varies from individual to individual. Therefore, the timing of pharmacological agents such as melatonin should be personalized according to the individual’s circadian rhythm. The implementation time and dose are also to be studied in the future [226].

### Neuroprotection and novel interventions

Animal studies have shown that rTMS has positive effects on neural regeneration and neuroprotection through inhibiting apoptotic cell death, as well as regulating neurotransmitters and neurotrophic factors [227]. Light therapy decreases oxidative stress markers [228] and removes Aβ via the lymphatic system of the brain [229] in AD mouse models, and reduces the loss of dopaminergic cells and increases tyrosine hydroxylase-positive cells in PD mouse models [230].

Orexin is a neuropeptide that contributes to the regulation of the sleep-wake cycle by increasing the arousal level and maintaining wakefulness. Suvorexant, one of the orexin receptor antagonists, has been approved to treat insomnia in elderly adults. Animal experiments showed that suvorexant can reduce amyloid-β plaques, improve synaptic plasticity, and restore the circadian phosphorylated CREB (cyclic AMP-response element binding protein) expression in the hippocampus of APP/PS1 mice [231].

Furthermore, some small-molecule modulators have been developed to restore the disrupted circadian system. For example, casein kinase 1 δ/ε inhibitor CKI-7 [232] significantly reduces  endogenous Aβ peptide production [233], thus playing an important role in neuroprotection. Rev-erbα is a core negative component of the circadian clock and modulates the cellular clock and energy metabolism [234]. Rev-erbα knock-out mice show disrupted diurnal patterns [235]. The agonists (GSK4112) and antagonists (SR8278) of Rev-erbα could correct the abnormal circadian rhythms [232], providing biological evidence for future trials of these small-molecule modulators as a therapeutic for neurodegeneration.

In addition, traditional Chinese herbs or herbal extracts and non-pharmacological interventions are proven beneficial to patients with neurdegenerative disorders, by exerting antioxidant and anti-inflammatory effects [236, 237]. Clinically, several studies showed that acupuncture [238] and Yang-Xue-Qing-Nao granules [239] improve sleep quality in PD patients. Future research should focus on the quality control of traditional Chinese medicine studies and figuring out the pharmacological mechanisms of main active ingredients.

## Conclusions and future prospect

Collectively, all behavioral, physiological and molecular aspects of circadian disturbances in neurodegenerative disorders provide substantial evidence that the circadian system is functionally impaired and most likely contributes to the deterioration of health and quality of life in patients inflicted with neurodegenerative diseases. However, although neurodegenerative disorders have overlapping circadian symptoms, the underlying neuro-pathophysiology may not necessarily be the same. Current evidence shows that dysfunction of the central SCN clock starts very early during the prodromal phase of AD, while the SCN itself functions normally till the early symptomatic phases where its dysfunction starts in both PD and HD [5].

Increasing research indicates that sleep dysfunction and circadian arrhythmicity are key aspects to consider when investigating neurodegenerative diseases. Although generally there are sleep and rhythmic disorders in different neurodegenerative diseases, each disease develops a specific phenotype to some extent [240]. For example, there is early impairment of the circadian homeostasis in AD, while in PD and HD, circadian homeostasis deterioration is more prevalent and occurs after diagnosis [240]. MSA and DLB have a higher prevalence of RBD. Our review reinforces the state of the field that bidirectional links of sleep and circadian rhythms with prevalent forms of neurodegeneration are likely.

Future research efforts are needed to center on the following fields. Despite great progress in understanding the basic mechanisms of the circadian clock and the neural circuitry of sleep, the knowledge of how these systems are affected in the brain in aging and neurodegenerative diseases is still rather superficial. It is necessary to fully characterize the putative bidirectional relationship of sleep and circadian circuits with neurodegeneration, in order to inform therapeutic targets. This will allow the field to expand the use of sleep and circadian rhythms as markers for early treatment of prodromal neurodegenerative disease and enable manipulation of the circadian system at the molecular and behavioral levels in longitudinal animal studies.

Systematically studying the circadian system in neurodegenerative diseases is another direction of future research, which calls for strict control of different circadian parameters, design of a set of evaluation tools, and development of personalized multi-component circadian interventions. Large longitudinal clinical studies are also needed to examine changes in circadian rhythms associated with progression of neurodegeneration and the relationship between different circadian markers and subsequent risk of developing neurodegenerative diseases, and to clarify whether circadian interventions for sleep disorders could prevent or delay the onset of neurodegenerative diseases.

Normalizing sleep and circadian disorders has the potential to reduce risk of neurodegeneration and improve quality of life and symptoms in those with neurodegenerative disorders. First, circadian-based interventions are a critical test of the hypothesis that circadian disruption is an integral component of the disease. Therefore, it is critical to establish a collaborative research program between clinical investigators and basic/translational neuroscientists, in order to advance the understanding of circadian regulation in neurodegeneration and effects of complex medication regimens on circadian function in animal models. Second, developing screen and therapeutic strategies in early (or “prodromal”) stage of neurodegenerative disease may facilitate earlier detection, prevention of disease progression and development of more effective therapeutic interventions. Third, optimization of existing therapies, such as light therapy and chronopharmacological principles, or launching novel neural circuit-based therapeutic interventions to restore the circadian activity, might provide beneficial effects against circadian alterations in patients with neurodegenerative disorders.

## Data Availability

Not applicable.

## Abbreviations

PD: Parkinson’s disease
AD: Alzheimer’s disease
HD: Huntington’s disease
SCN: Suprachiasmatic nucleus
ipRGC: Intrinsically photoreceptive retinal ganglion cell
PVN: Paraventricular nucleus
MPN: Medial preoptic nucleus
TTFL: Transcription-translation feedback loops
BMAL1: Brain and muscle Arnt-like protein 1
CLOCK: Circadian locomotor output cycles kaput
CRY: Cryptochrome
EEG: Electroencephalography
EMG: Electromyography
REM: Rapid eye movement
NREM: Non-REM
RSWA: REM sleep without atonia
RBD: REM sleep behavior disorder
RLS: Restless leg syndrome
SDB: Sleep-disordered breathing
EDS: Excessive daytime sleepiness
DEB: Dream-enacting behaviors
OSA: Obstructive sleep apnea
AHI: Apnea hypopnea index
HRV: Heart rate variability
CBT: Core body temperature
MSA: Multiple system atrophy
SWS: Slow wave sleep
VIP: Vasoactive intestinal peptide
DLB: Dementia with lewy bodies
FTD: Frontotemporal dementia
iRBD: Isolated RBD
MCI: Mild cognitive impairment
pRBD: Probable RBD
PSG: Polysomnography
SRMD: Sleep-related movement disorders
BLT: Bright light therapy
rTMS: Repetitive transcranial magnetic stimulation
tDCS: Transcranial direct current stimulation
